# Secure Wireless Communication for Correlated Legitimate User and Eavesdropper Channels via Movable-Antenna Enhanced Frequency Diverse Array

**DOI:** 10.3390/e27040401

**Published:** 2025-04-09

**Authors:** Xuehan Wu, Huaizong Shao, Jingran Lin, Ye Pan, Weijie Xiong

**Affiliations:** 1 School of Information and Communication Engineering, University of Electronic Science and Technology of China, Chengdu 611731, China; xhwu@std.uestc.edu.cn (X.W.); hzshao@uestc.edu.cn (H.S.); pany@uestc.edu.cn (Y.P.); 202311012313@std.uestc.edu.cn (W.X.); 2 Laboratory of Electromagnetic Space Cognition and Intelligent Control, Beijing 100083, China; 3 Tianfu Jiangxi Laboratory, Chengdu 641419, China

**Keywords:** movable antenna (MA), frequency diverse arrays (FDA), physical-layer (PHY) security, transmit beamforming

## Abstract

Physical-layer (PHY) security is widely used as an effective method for ensuring secure wireless communications. However, when the legitimate user (LU) and the eavesdropper (Eve) are in close proximity, the channel coupling can significantly degrade the secure performance of PHY. Frequency diverse array (FDA) technique addresses channel coupling issues by introducing frequency offsets among array elements. However, FDA’s ability to secure communication relies mainly on frequency domain characteristics, lacking the spatial degrees of freedom. The recently proposed movable antenna (MA) technology serves as an effective approach to overcome this limitation. It offers the flexibility to adjust antenna positions dynamically, thereby further decoupling the channels between LU and Eve. In this paper, we propose a novel MA-FDA approach, which offers a comprehensive solution for enhancing PHY security. We aim to maximize the achievable secrecy rate through the joint optimization of all antenna positions at the base station (BS), FDA frequency offsets, and beamformer, subject to the predefined regions for antenna positions, frequency offsets range, and energy constraints. To solve this non-convex optimization problem, which involves highly coupled variables, the alternating optimization (AO) method is employed to cyclically update the parameters, with the projected gradient ascent (PGA) method and block successive upper-bound minimization (BSUM) method being employed to tackle the challenging subproblems. Simulation results demonstrate that the MA-FDA approach can achieve a higher secrecy rate compared to the conventional phased array (PA) or fixed-position antenna (FPA) schemes.

## 1. Introduction

Physical-layer (PHY) security techniques have been extensively researched as an effective approach to achieve secure wireless communications [1]. Unlike traditional cryptographic methods, PHY security techniques provide confidentiality at the PHY level without relying on mathematical encryption [2]. Transmit beamforming is one of the most widely used methods for ensuring PHY security [3,4,5]. The security is achieved by improving the reception quality for legitimate user (LU) or jamming the reception of eavesdropper (Eve) through beamforming [6]. However, when the LU and the Eve are in close proximity, the angle of them are nearly the same. In such cases, conventional beamforming techniques, such as phased array (PA), encounter significant challenges, as the LU and Eve experience highly correlated channels [7], since the beampattern of conventional beamforming techniques is angle-dependent.

In contrast, the characteristics of frequency diverse array (FDA) are inherently suited to addressing channel coupling issues [8]. FDA introduces small frequency offsets across the array elements, resulting in cumulative phase lags among the array antennas [9]. Unlike the fixed lags in PA, FDA generates an angle–range–time-dependent beampattern, which facilitates channel decoupling between LU and Eve when their angles are similar [10]. By exploiting this capability, we can effectively differentiate the received signal levels of the LU and Eve, even when they are geographically close. However, the array configuration of FDA is fixed, limiting the system’s ability to dynamically adjust spatial correlation across different steering angles. This spatial limitation reduces its effectiveness in maintaining a high secrecy rate, as the spatial correlation between desired and undesired users cannot be easily mitigated by altering the antenna positions. To overcome this challenge, the recently proposed movable antenna (MA) technology is employed in this system. It is an effective method to utilize channel variation in a continuous spatial field [11]. Specifically, an MA is connected to the radio frequency (RF) chain via a flexible cable [12]. By allowing the flexible adjustment of antenna positions within a specified area, MA introduces an additional spatial degree of freedom [13]. This flexibility enables the modification of steering vectors for different angles, reconfiguring wireless channels to further decouple them and enhance PHY security [14]. FDA decouples the channels of the LU and Eve by the frequency domain capabilities, while MA achieves decoupling by leveraging spatial flexibility. By employing both FDA and MA technologies, it is possible to tackle the channel coupling challenges through multidimensional channel reconstruction.

This paper explores the utilization of the MA-FDA approach to enhance secure communication from an MA-FDA-enabled base station (BS) to a single-antenna user, considering the presence of a single-antenna eavesdropper. We aim to maximize the achievable secrecy rate through the joint optimization of antenna positions at the BS, the FDA frequency offsets and beamformer. An alternating optimization (AO) algorithm is developed to solve the formulated highly non-convex problem.

The main contributions of this work can be summarized as follows:

- We propose the idea of employing MA and FDA to achieve PHY security. FDA can decouple the channels between the proximal LU and Eve through frequency domain capabilities. MA further enhances the decoupling performance by providing spatial degrees of freedom that compensate for the limitations of FDA. The proposed MA-FDA approach maximizes PHY security through multidimensional channel reconstruction, thereby achieving a higher secrecy rate.
- To address this idea, we formulate a secrecy rate maximization problem by jointly optimizing the positions of antennas, FDA frequency offsets, and beamforming vectors, subject to the predefined regions for antenna positions, frequency offsets range and energy constraints. The proposed formulation fully exploits the spatial and frequency degrees of freedom in the MA-FDA system, thereby significantly enhancing PHY security.
- The secrecy rate maximization problem is a non-convex problem involving coupled variables. To solve this difficult problem, the proposed problem is partitioned into several subproblems. An algorithm is then designed using the AO method [15] to iteratively update the parameters, specifically the antenna positions, FDA frequency offsets, and beamformers. However, as these subproblems remain difficult to solve, we employ the projected gradient ascent (PGA) method and block a successive upper-bound minimization (BSUM) method to handle them.

### 1.1. Related Works

Previous studies have highlighted the significant potential of MA array-enabled communications [16]. The authors in [17] demonstrated that the angle estimation performance in wireless sensing is fundamentally dictated by array geometry and proposes a system with a MA array, allowing flexible antenna positioning to enhance performance. Ref. [18] introduces MA technology to wireless surveillance for the first time and proposes two schemes to determine locally optimal and suboptimal antenna positions. Ref. [19] proposes an RIS-assisted MA system to enhance the sum-rate performance and employs an adaptive fractional programming quadratic transform algorithm based on a genetic algorithm (FPQT-GA) to analyze the system. In [12], the authors investigated the MA-enhanced multiple-access channel (MAC) for the uplink transmission from multiple users and optimizes it using the proposed multi-directional descent (MDD) framework. Additionally, several related studies in the field of secure communications are worth mentioning. In [20], the authors proposed a joint optimization of beamforming and MA positioning at the BS to minimize total transmit power, subject to each user meeting the required minimum signal-to-interference-plus-noise ratio (SINR). In [21], a novel MA-enhanced multiple-input–multiple-output (MIMO) system was proposed to leverage the associated spatial degrees of freedom to improve wireless communication performance. Additionally, there are a few related works on MA-based secure communication. In [22], MA array-assisted PHY security was achieved through jointly optimizing the transmit beamforming and positions of all antennas. Ref. [23] proposed an MA-enabled secure transmission scheme with power consumption minimization and secrecy rate maximization. In [24], a covert communication system empowered by MA array is proposed, and the transmit beamforming and antenna position design problem is solved by an alternative algorithm. However, these works did not address the case that the channels of the LU and Eve exhibit a strong correlation, such as when the LU and Eve are in close proximity.

The angle–range–time-dependent characteristics of FDA can be employed for PHY secure communication [25,26,27]. Several studies on FDA-based secure communication are noteworthy.The authors in [28,29,30] studied the artificial noise (AN)-aided techniques that impose AN on Eve without adversely affecting the signal-to-noise-ratio (SNR) at LU. In [31], the authors employed FDA to develop an orthogonal frequency-division multiplexing (OFDM) transmitter that has the capability to achieve PHY security. Direction-dependent communications proposed in [32] used two-bit phase control across a two-element FDA antenna, which offers PHY security for wireless communications. In [33], a covert wireless communication scheme was proposed utilizing a random frequency diverse array, considering two scenarios in which the user’s location is either known or unknown to eavesdropper. In [34], the authors proposed a 3D directional modulation (DM) scheme utilizing randomized radiation with FDSA to enhance PLS. The approach employs a planar array, and a genetic algorithm (GA)-based optimization strategy is applied. However, the use of FDA with fixed-position antenna (FPA) still limits secure communication performance, due to the inherent spatial correlation across different steering angles [22]. Consequently, the proposed MA-FDA approach addresses the significant channel coupling challenges of proximal LU and Eve case, which is completely different from existing works.

### 1.2. Organization and Notations

The remainder of this paper is organized as follows. In Section 2, we describe the MA-FDA system model, and formulate the secrecy rate maximization problem. The proposed AO algorithm is presented in Section 3. Section 4 provides numerical simulations to validate the efficacy of the proposed AO algorithm. Finally, we conclude this paper in Section 5.

Notations: Italic letters represent scalars, Boldface uppercase denote matrices, and lowercase letters indicate vectors. ${\left(\cdot \right)}^{†}$ and ${\left(\cdot \right)}^{H}$ represent transpose and Hermitian of (·), respectively. $C^{M\times N}$ and $R^{M\times N}$ are used to indicate complex and real M×N matrices, respectively. ⌊·⌋ and ⌈·⌉ round the argument to the nearest integer towards −∞ and *∞*, respectively.

## 2. System Model and Problem Statement

### 2.1. System Model

**Figure 1:**
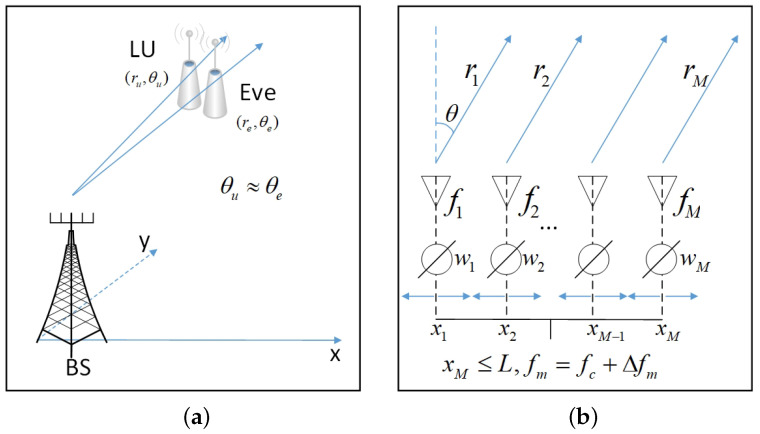
System model: (**a**) Close-proximity LU and Eve; (**b**) MA-FDA-based beamforming for PHY security.

We consider a secure transmission system consisting of an MA-FDA-enabled BS, a single-antenna LU, and a single-antenna Eve, as shown in Figure 1. The position of Eve can be obtained through sensing techniques such as beamforming scanning and direction estimation. To enhance the quality of secure communications, the BS employs a multi-antenna linear MA and FDA technologies to ensure the secure transmission of confidential information to the LU despite the presence of Eve.

The position of the *m*th antenna at BS is defined as $x_{m},m=1,2,\ldots ,M$, where M denotes the total number of antennas. Unlike traditional PA, FDA utilizes small frequency offsets ${\left\{\Delta f_{m}\right\}}_{m=1}^{M}$ across the array antennas. We assume that $0\leq \Delta f_{m}\leq \Delta F$ and $\Delta F\ll f_{c}$, with ΔF and $f_{c}$ representing the frequency offsets upper bound and carrier frequency, respectively. As illustrated in Figure 1, the radiation frequency for the *m*th antenna is $f_{m}=f_{c}+\Delta f_{m},m=1,2,\ldots ,M$. To prevent aliasing effects, the distance between antennas should satisfy $d_{m}\geq d_{m,min}=c/\left(2f_{m}\right)$ for frequency $f_{m}$, where *c* represents the speed of light in free space. Taking comprehensive consideration, the upper bound of antenna spacing is $d_{min}=\min_{m}d_{m}=c/\left[2\left(f_{c}+\max_{m}\Delta f_{m}\right)\right]\approx c/\left(2f_{c}\right)$. Consequently, we deploy a linear MA array with an applicable antenna space, i.e., $\left|x_{m}-x_{k}\right|\geq d_{min}=c/\left(2f_{c}\right),1\leq m\neq k\leq M$.

Without loss of generality, we assume that the first antenna is positioned at the origin of the coordinate system. The response of the directional channel associated with the *m*th FDA antenna for the receiver in position (r,θ) at time *t* can be expressed as [35]

(1) $$h_{m}\left(f_{m},r,\theta ,t,x_{m}\right)=e^{-j2\pi f_{m}\left[t-\frac{r-x_{m}sin\left(\theta \right)}{c}\right]},$$

The FDA beampattern at time *t* is calculated by combining the channel responses of the *M* elements,

(2) $$\begin{array}{cc} B(f,r,\theta ,t,x_{m}) & =\sum_{m=1}^{M}h_{m}\left(f_{m},r,\theta ,t,x_{m}\right)\\ \; & =\sum_{m=1}^{M}e^{-j2\pi f_{m}\left[t-\frac{r-x_{m}sin\left(\theta \right)}{c}\right]}\\ \; & =e^{-j2\pi f_{c}t}\sum_{m=1}^{M}e^{j[\Phi_{m}^{0}\left(r,\theta ,x_{m}\right)+\Phi_{m}^{1}\left(\Delta f_{m},r,\theta ,t,x_{m}\right)]}. \end{array}$$

where $\Phi_{m}^{0}\left(r,\theta ,x_{m}\right)=2\pi f_{c}\frac{r-x_{m}sin\left(\theta \right)}{c}$, $\Phi_{m}^{1}\left(\Delta f_{m},r,\theta ,t,x_{m}\right)=-2\pi \Delta f_{m}\left[t-\frac{r-x_{m}sin\left(\theta \right)}{c}\right]$, ∀m, and $f≜{\left(f_{1},f_{2},\ldots ,f_{M}\right)}^{†}$.

It can be seen from Equation (2) that FPA is a particular case of the MA under consideration, i.e., when antenna positions ${\left\{x_{m}\right\}}_{m=1}^{M}$ are fixed, MA reduces to traditional FPA, consequently generating inherent spatial correlation across different angles. The flexibility of ${\left\{x_{m}\right\}}_{m=1}^{M}$ introduces additional spatial degrees of freedom, enabling better spatial diversity and multiplexing performance, thus having the potential to achieve a higher secrecy rate. Additionally, considering the characteristic imparted by the frequency offsets ${\left\{\Delta f_{m}\right\}}_{m=1}^{M}$ of FDA, Equation (2) indicates that ${\left\{\Phi_{m}^{1}\left(\Delta f_{m},r,\theta ,t\right)\right\}}_{m=1}^{M}$ depends on the frequency offsets ${\left\{\Delta f_{m}\right\}}_{m=1}^{M}$, the location (r,θ), and the time *t*, besides ${\left\{x_{m}\right\}}_{m=1}^{M}$. Consequently, the FDA beampattern exhibits angle–range–time-dependence for non-zero frequency offsets ${\left\{\Delta f_{m}\right\}}_{m=1}^{M}$ due to the distinct accumulating phase lags among different antennas. When $\Delta f_{m}=0$ for all *m*, FDA reverts to traditional PA, which is range–time-independent. Consequently, it is unable to effectively distinguish between the LU and Eve when their angles are similar, i.e., $\theta_{u}\approx \theta_{e}$, where $\theta_{u}$ and $\theta_{e}$ represent the angles of LU and Eve, respectively, due to the strong correlation between channels. By introducing frequency offsets, the strong correlation between the channels of LU and Eve is mitigated. In the FDA beampattern, LU and Eve are effectively separated, thereby having the capacity to enhance PHY security. However, it is noteworthy that the selection of appropriate antenna positions and frequency offsets to ensure secure communication is a significant challenge. The beampattern peak and valley are influenced by antenna positions and frequency offsets, as shown in the complex Equation (2). Random choice often lead to unsatisfactory results, thus necessitating a deliberate selection of antenna positions and frequency offsets.

### 2.2. Problem Formulation

In order to fully explore the potential of the antenna array in ensuring PHY security, a novel MA-FDA beamforming strategy is devised by integrating MA with FDA. It should be emphasized that the time-dependent characteristics of the FDA channel and beampattern result in a time-varying beamforming solution, including the transmit beamformer.

The digital transmit beamforming of the BS for the confidential information at time *t* is defined as $w\left(t\right)≜{\left[w_{1}\left(t\right),w_{2}\left(t\right),\ldots ,w_{M}\left(t\right)\right]}^{†}\in C^{M\times 1}$. The channel vector between the array and the receiver is $h\left(f,r,\theta ,t,x\right)=[h_{1}\left(f_{1},r,\theta ,t,x_{1}\right),h_{2}\left(f_{2},r,\theta ,t,x_{2}\right),\ldots$, $h_{M}\left(f_{M},r,\theta ,t,x_{M}\right){]}^{†}\in C^{M\times 1}$, where $x={\left[x_{1},x_{2},\ldots ,x_{M}\right]}^{†}\in R^{M\times 1}$. The SNR of the MA-FDA at time *t* is derived as

(3) $$\begin{array}{c} \mathrm{SNR}\left(f,x,w\right)=\sigma^{-2}{\left|h^{H}\left(f,r,\theta ,t,x\right)w\left(t\right)\right|}^{2}. \end{array}$$

Define $\left(r_{u},\theta_{u}\right)$ and $\left(r_{e},\theta_{e}\right)$ as the coordinates of the proximal LU and Eve, respectively. For convenience, we denote $h_{u}\left(f,t,x\right)$ and $h_{e}\left(f,t,x\right)$ as the response of the corresponding channels, i.e., $h_{u}\left(f,t,x\right)≜h\left(f,r_{u},\theta_{u},t,x\right)$ and $h_{e}\left(f,t,x\right)≜h\left(f,r_{e},\theta_{e},t,x\right)$. For the signal transmitted at time *t*, the achievable rates at LU and Eve are defined as

(4a) $$\begin{array}{c} R_{u}\left(f,x,w\right)=\log(1+\sigma_{u}^{-2}|h_{u}^{H}\left(f,t,x\right)w\left(t\right){|}^{2}), \end{array}$$

(4b) $$\begin{array}{c} R_{e}\left(f,x,w\right)=\log(1+\sigma_{e}^{-2}|h_{e}^{H}\left(f,t,x\right)w\left(t\right){|}^{2}), \end{array}$$

where $\sigma_{u}$ and $\sigma_{e}$ are the noise powers corresponding to LU and Eve, respectively. Based on Equations (4a) and (4b), the achievable secrecy rate is given by

(5) $$\begin{array}{c} R_{\sec}\left(f,x,w\right)={\left[\begin{array}{c} \log(1+\sigma_{u}^{-2}|h_{u}^{H}\left(f,t,x\right)w\left(t\right){|}^{2})\\ -\log(1+\sigma_{e}^{-2}|h_{e}^{H}\left(f,t,x\right)w\left(t\right){|}^{2}) \end{array}\right]}^{+}, \end{array}$$

where ${\left[\cdot \right]}^{+}=\max\left(\cdot ,0\right)$. When $R_{\sec}\left(f,x,w\right)<0$, the system stops information transmission. Therefore, in the following analysis, we focus on the case that $R_{\sec}\left(f,x,w\right)>0$, i.e., the communication quality of LU is better than Eve.

We aim to maximize the secrecy rate through the joint optimization of the positions of the MA ${\left\{x_{m}\right\}}_{m=1}^{M}$, FDA frequency offsets ${\left\{\Delta f_{m}\right\}}_{m=1}^{M}$ and the transmit beamformer w. Consequently, the secrecy rate maximization problem leveraging MA-FDA beamforming can be expressed as

(6a) $$\begin{array}{cc} \max_{f,x,w}\; & R_{\sec}\left(f,x,w\right) \end{array}$$

(6b) $$\begin{array}{cc} s.t.\;\; & |x_{m}-x_{k}|\geq d_{min},1\leq m\neq k\leq M \end{array}$$

(6c) $$\begin{array}{cc} \; & {\left\{x_{m}\right\}}_{m=1}^{M}\in \left[0,L\right], \end{array}$$

(6d) $$\begin{array}{cc} \; & f_{c}-\Delta F\leq f_{m}\leq f_{c}+\Delta F,\;\forall \;m, \end{array}$$

(6e) $$\begin{array}{cc} \; & {∥w∥}_{2}^{2}\leq P, \end{array}$$

where [0,L] is the range of MA, and L≥(M−1)d must be satisfied, *P* is the power budget of BS. In the next section, an optimization algorithm is proposed to address the non-convex problem (6a)–(6e).

## 3. AO Algorithm for Problem (6a)–(6e)

Problem (6a)–(6e) is a challenge due to the beampattern gain being a complex non-convex function with coupled variables, namely f, x, w.

It is important to note that constraints (6b) and (6c) only involve the variable x for the antenna position optimization, constraint (6d) involves only the variable f for frequency offsets optimization, and constraint (6e) involves only the variable w for FDA beamformer design. Consequently, we can divide the optimization variables into three separate components, i.e., {f}, {x}, and {w}. By employing this division, an iterative algorithm can be designed to solve the problem (6a)–(6e). Specifically, we optimize problem (6a)–(6e) by iteratively solving three subproblems: i.e., update one of the three blocks while keeping the other two fixed, which are referred to as the {f}-subproblem, {x}-subproblem, and {w}-subproblem, respectively. This approach falls within the framework of the AO algorithm.

### 3.1. {f}-Subproblem

We update the frequency offsets f with given x and w.

(7) $$\begin{array}{cc} \max_{f}\; & R_{\sec}\left(f\right)\\ s.t.\;\; & (6d)\;\mathrm{is}\;\mathrm{satisfied}. \end{array}$$

According to reference [36], the optimal secrecy rate for the multi-input, single-output, single-eavesdropper (MISOSE) secrecy design problem can be obtained using the method of generalized eigendecomposition, i.e.,

(8) $$\begin{array}{c} R_{\sec}^{*}\left(f\right)=\log\left(1+\max\left(0,\lambda \left(f,t\right)\right)\right), \end{array}$$

where λ(f,t) is the principal eigenvalue of Ψ(f,t), and $\Psi \left(f,t\right)=\left(H_{e}^{-\frac{1}{2}}\left(f,t\right)+\frac{1}{P}I\right)\left[H_{u}\left(f,t\right)-H_{e}\left(f,t\right)\right]\left(H_{e}^{-\frac{1}{2}}\left(f,t\right)+\frac{1}{P}I\right)$, $H_{u}\left(f,t\right)=\sigma_{u}^{-2}h_{u}\left(f,t,x\right)h_{u}^{†}\left(f,t,x\right)$, $H_{e}\left(f,t\right)=\sigma_{e}^{-2}h_{e}\left(f,t,x\right)h_{e}^{†}\left(f,t,x\right)$. Therefore, maximizing the principal eigenvalue λ(f,t) enables the achievement of PHY security. As a result, problem (7) is equivalent to the following formulation:

(9) $$\begin{array}{cc} \max_{f}\; & \lambda (f,t)\\ s.t.\;\; & (6d)\;\mathrm{is}\;\mathrm{satified}. \end{array}$$

By analyzing the structure of the matrix Ψ(f,t), we find that problem (9) is equivalent to decorrelating the channels of LU and Eve, represented by $|h_{e}^{†}\left(f,t\right)h_{u}{\left(f,t\right)|}^{2}$. Consequently, the corresponding optimization problem for f can be formulated as

(10) $$\begin{array}{cc} \min_{f}\; & |h_{e}^{H}\left(f,t\right)h_{u}{\left(f,t\right)|}^{2}\\ s.t.\;\; & (6d)\;\mathrm{is}\;\mathrm{satisfied}. \end{array}$$

And the objective can be written as

(11) $$\begin{array}{cc} {\left|h_{e}^{H}\left(f,t\right)h_{u}\left(f,t\right)\right|}^{2} & ={\left|\sum_{m=1}^{M}e^{j2\pi (f_{m}\frac{\left(m-1\right)x_{m}\left(\sin\theta_{e}-\sin\theta_{u}\right)}{c}))}\right|}^{2}\\ \; & =\sum_{m=1}^{M}\sum_{n=1}^{N}e^{j2\pi (f_{m}\tau_{m}-f_{n}\tau_{n})}, \end{array}$$

where $\tau_{m}=\frac{\left(m-1\right)x_{m}\left(\sin\theta_{e}-\sin\theta_{u}\right)}{c},\forall m$. Utilizing Euler’s formula, the sum of two conjugate complex exponential terms can be reformulated as the sum of two cosine terms, i.e.,

(12) $$\begin{array}{c} {\left|h_{e}^{H}\left(f,t\right)h_{u}\left(f,t\right)\right|}^{2}=\sum_{m=1}^{M}\sum_{n=1}^{N}\cos\left[2\pi (f_{m}\tau_{m}-f_{n}\tau_{n})\right]. \end{array}$$

The corresponding problem is finally expressed as

(13) $$\begin{array}{cc} \min_{f}\; & \sum_{m=1}^{M}\sum_{n=1}^{N}\cos\left[2\pi (f_{m}\tau_{m}-f_{n}\tau_{n})\right]\\ s.t.\;\; & (6d)\;\mathrm{is}\;\mathrm{satified}, \end{array}$$

Problem (13) is challenging due to its non-convex objective function. By exploiting the separability in Equation (6d), the subproblem of f can be further divided into *M* smaller problems, each associated with updating $f_{m},\;for\;m=1,2,\ldots ,M$, respectively. According to the BSUM framework, each block is firstly approximated by a tight upper bound, and then optimized iteratively, with the other blocks held constant.

Without loss of generality, we focus on updating $f_{m}$ during the *i*th iteration. By fixing the remaining frequency variables, i.e., $f_{n},\forall n\neq m$, we have the corresponding subproblem,

(14) $$\begin{array}{cc} \min_{f_{m}}\; & \sum_{\begin{array}{c} n=1\\ n\neq m \end{array}}^{M}\cos\left[2\pi \left(f_{m}\tau_{m}-f_{n}^{i-1}\tau_{n}\right)\right]\\ s.t.\;\; & (6d)\;\mathrm{is}\;\mathrm{satified}, \end{array}$$

where $f_{n}^{i-1}$ denotes the value of $f_{n}$ in the previous iteration, with the superscript indicates the number of iterations.

The non-concave objective in problem (14) should be approximated by a tight upper bound. To this end, we first express the objective in problem (14) as

(15a) $$\begin{array}{cc} \; & g_{m}\left(f_{m};f_{-m}^{i-1}\right)≜\sum_{\begin{array}{c} n=1\\ n\neq m \end{array}}^{M}{\tilde{g}}_{m,n}\left(f_{m};f_{n}^{i-1}\right), \end{array}$$

(15b) $$\begin{array}{cc} \; & {\tilde{g}}_{m,n}\left(f_{m};f_{n}^{i-1}\right)≜\cos\left[2\pi \left(f_{m}\tau_{m}-f_{n}^{i-1}\tau_{n}\right)\right],n\neq m, \end{array}$$

where $f_{-m}^{i-1}≜{\left[f_{1}^{i-1},\ldots ,f_{m-1}^{i-1},f_{m+1}^{i-1},\ldots ,f_{M}^{i-1}\right]}^{†}$ represents the remaining subvector of $f^{i-1}$ after deleting $f_{m}^{i-1}$ from it. Next, the non-concave cos(·) function in ${\tilde{g}}_{m,n}\left(f_{m};f_{n}^{i-1}\right)$ is approximated using a concave quadratic function ${\tilde{\eta }}_{m,n}\left(f_{m};f_{m}^{i-1},f_{n}^{i-1}\right)$, which is expressed as

(16a) $$\begin{array}{cc} \eta_{m}\left(f_{m};f^{i-1}\right) & ≜\sum_{\begin{array}{c} n=1\\ n\neq m \end{array}}^{M}{\tilde{\eta }}_{m,n}\left(f_{m};f_{m}^{i-1},f_{n}^{i-1}\right), \end{array}$$

(16b) $$\begin{array}{cc} {\tilde{\eta }}_{m,n}\left(f_{m};f_{m}^{i-1},f_{n}^{i-1}\right) & ≜\kappa_{m,n}{\left(f_{m}-\zeta_{m,n}\right)}^{2}+\delta_{m,n},n\neq m, \end{array}$$

where $\kappa_{m,n}\in R_{-}$, $\zeta_{m,n}\in R$, and $\delta_{m,n}\in R$, n≠m, are the parameters of the quadratic function which must be carefully selected to ensure that $\eta_{m}\left(f_{m};f^{i-1}\right)$ serves as a tight upper bound of $g_{m}\left(f_{m};f_{-m}^{i-1}\right)$. To this end, we require that

(17a) $$\begin{array}{cc} \; & {\tilde{\eta }}_{m,n}\left(f_{m}^{i-1};f_{m}^{i-1},f_{n}^{i-1}\right)={\tilde{g}}_{m,n}\left(f_{m}^{i-1};f_{n}^{i-1}\right), \end{array}$$

(17b) $$\begin{array}{cc} \; & {\tilde{\eta }}_{m,n}^{\prime }\left(f_{m}^{i-1};f_{m}^{i-1},f_{n}^{i-1}\right)={\tilde{g}}_{m,n}^{\prime }\left(f_{m}^{i-1};f_{n}^{i-1}\right), \end{array}$$

(17c) $$\begin{array}{cc} \; & {\tilde{g}}_{m,n}\left(\zeta_{m,n};f_{n}^{i-1}\right)\geq {\tilde{g}}_{m,n}\left(f_{m}^{i-1};f_{n}^{i-1}\right), \end{array}$$

(17d) $$\begin{array}{cc} \; & {\tilde{g}}_{m,n}\left(\zeta_{m,n};f_{n}^{i-1}\right)\in \left\{1,-1\right\}, \end{array}$$

(17e) $$\begin{array}{cc} \; & \left|\zeta_{m,n}-f_{m}^{i-1}\right|<\frac{1}{2\left|\tau_{m}\right|}, \end{array}$$

where (17a) and (17b) ensure that ${\tilde{\eta }}_{m,n}\left(f_{m}^{i-1};f_{m}^{i-1},f_{n}^{i-1}\right)$ and ${\tilde{g}}_{m,n}\left(f_{m}^{i-1};f_{n}^{i-1}\right)$ are tight with each other and have the same derivative at $f_{m}=f_{m}^{i-1}$; (17c) guarantees the upper bound condition; (17d) demonstrates that the quadratic function and the cos(·) function such that ${\tilde{\eta }}_{m,n}\left(f_{m}^{i-1};f_{m}^{i-1},f_{n}^{i-1}\right)$ is the optimal quadratic upper bound of ${\tilde{g}}_{m,n}\left(f_{m}^{i-1};f_{n}^{i-1}\right)$; (17e) restricts the distance between $f_{m}^{i-1}$ and $\zeta_{m,n}$ to further satisfy the upper bound requirement.

Adhering to these constraints, $\left\{\kappa_{m,n},\zeta_{m,n},\delta_{m,n}\right\}$ should be selected based on the value of ${\tilde{g}}_{m,n}^{\prime }\left(f_{m}^{i-1};f_{n}^{i-1}\right)≜-2\pi \tau_{m}\sin\left[2\pi \left(f_{m}^{i-1}\tau_{m}-f_{n}^{i-1}\tau_{n}\right)\right]$. Specifically, the solution can be expressed as

(18a) $$\begin{array}{cc} \; & \mathrm{if}\;\;{\tilde{g}}_{m,n}^{\prime }\left(f_{m}^{i-1};f_{n}^{i-1}\right)=0\\ \; & \;\;\;\kappa_{m,n}=-\left(1+\delta_{m,n}\right)\pi^{2}\tau_{m}^{2},\\ \; & \;\;\;\zeta_{m,n}=f_{m}^{i-1},\\ \; & \;\;\;\delta_{m,n}=\cos\left[2\pi \left(f_{m}^{i-1}\tau_{m}-f_{n}^{i-1}\tau_{n}\right)\right]\in \left\{1,-1\right\}, \end{array}$$

(18b) $$\begin{array}{cc} \; & \mathrm{else}\\ \; & \;\;\;\kappa_{m,n}=\frac{-\pi \tau_{m}\sin\left[2\pi \left(f_{m}^{i-1}\tau_{m}-f_{n}^{i-1}\tau_{n}\right)\right]}{f_{m}^{i-1}-\zeta_{m,n}},\\ \; & \;\;\;\zeta_{m,n}=\left\{\begin{array}{cc} \; & \frac{\left\lceil (f_{m}^{i-1}\tau_{m}-f_{n}^{i-1}\tau_{n})\right\rceil +f_{n}^{i-1}\tau_{n}}{\tau_{m}},\\ \; & \;\mathrm{if}\;{\tilde{g}}_{m,n}^{\prime }\left(f_{m}^{i-1};f_{n}^{i-1}\right)>0,\\ \; & \frac{\left\lfloor (f_{m}^{i-1}\tau_{m}-f_{n}^{i-1}\tau_{n})\right\rfloor +f_{n}^{i-1}\tau_{n}}{\tau_{m}},\\ \; & \;\mathrm{if}\;{\tilde{g}}_{m,n}^{\prime }\left(f_{m}^{i-1};f_{n}^{i-1}\right)<0, \end{array}\right.\\ \; & \;\;\;\delta_{m,n}=\cos\left[2\pi \left(f_{m}^{i-1}\tau_{m}-f_{n}^{i-1}\tau_{n}\right)\right]-\kappa_{m,n}{\left(f_{m}^{i-1}-\zeta_{m,n}\right)}^{2}, \end{array}$$

**Figure 2:**
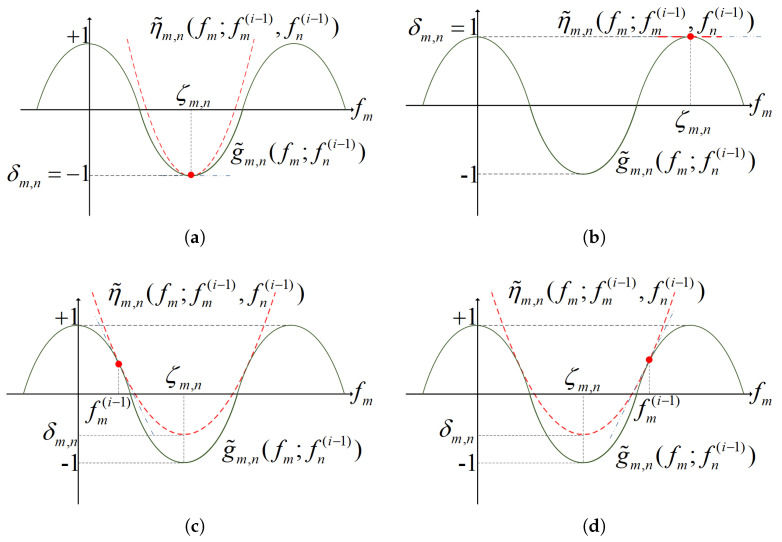
Use a concave quadratic function to approximate cos(·).

It is more clearly illustrated in Figure 2. There are four cases in which the cos(·) is approximated:

- Case 1: When ${\tilde{g}}_{m,n}^{\prime }\left(f_{m}^{i-1};f_{n}^{i-1}\right)=0$ and ${\tilde{g}}_{m,n}\left(f_{m}^{i-1};f_{n}^{i-1}\right)=-1$ as shown in Figure 2a, ${\tilde{\eta }}_{m,n}\left(f_{m};f_{m}^{i-1},f_{n}^{i-1}\right)=1$ is a straight line;
- Case 2: When ${\tilde{g}}_{m,n}^{\prime }\left(f_{m}^{i-1};f_{n}^{i-1}\right)=0$ and ${\tilde{g}}_{m,n}\left(f_{m}^{i-1};f_{n}^{i-1}\right)=1$ as shown in Figure 2b, ${\tilde{\eta }}_{m,n}\left(f_{m};f_{m}^{i-1},f_{n}^{i-1}\right)$ is formed as a quadratic function, symmetrical along $f_{m}^{i-1}$;
- Case 3: When ${\tilde{g}}_{m,n}^{\prime }\left(f_{m}^{i-1};f_{n}^{i-1}\right)<0$ as shown in Figure 2c, ${\tilde{\eta }}_{m,n}\left(f_{m};f_{m}^{i-1},f_{n}^{i-1}\right)$ is formed as a quadratic function, which is symmetric about the first-right valley point of ${\tilde{g}}_{m,n}\left(f_{m}^{i-1};f_{n}^{i-1}\right)$ beside the tangential point;
- Case 4: When ${\tilde{g}}_{m,n}^{\prime }\left(f_{m}^{i-1};f_{n}^{i-1}\right)>0$ as shown in Figure 2d, ${\tilde{\eta }}_{m,n}\left(f_{m};f_{m}^{i-1},f_{n}^{i-1}\right)$ is formed as a quadratic function, which is symmetric about the first-left valley point of ${\tilde{g}}_{m,n}\left(f_{m}^{i-1};f_{n}^{i-1}\right)$ beside the tangential point.

By substituting $g_{m}\left(f_{m};f_{-m}^{i-1}\right)$ with $\eta_{m}\left(f_{m};f^{i-1}\right)$, problem (14) is approximated by

(19) $$\begin{array}{cc} \min_{f_{m}}\; & \sum_{\begin{array}{c} n=1\\ n\neq m \end{array}}^{M}\left[\kappa_{m,n}{\left(f_{m}-\zeta_{m,n}\right)}^{2}+\delta_{m,n}\right]\\ s.t.\;\; & (6d)\;\mathrm{is}\;\mathrm{satified}, \end{array}$$

which exhibits strong concavity, and thus $f_{m}$ has a unique solution given by

(20) $$f_{m}={\left[\frac{\sum_{\begin{array}{c} n=1\\ n\neq m \end{array}}^{M}\kappa_{m,n}\zeta_{m,n}}{\sum_{\begin{array}{c} n=1\\ n\neq m \end{array}}^{M}\kappa_{m,n}}\right]}_{f_{c}-\Delta F}^{f_{c}+\Delta F},$$

where ${\left[\;\cdot \;\right]}_{f_{c}-\Delta F}^{f_{c}+\Delta F}$ represents the projection onto $\left[f_{c}-\Delta F,\;f_{c}+\Delta F\right]$. The complexity of the proposed approach is low, as each step involves a simple closed-form solution.

### 3.2. {x}-Subproblem

To facilitate the subsequent analysis, we adopt the real-valued form for the construction of our algorithm. The real-valued channel corresponding to the *m*th FDA antenna is defined as

(21a) $$\begin{array}{cc} k_{m} & ≜\mathrm{real}\left(h_{m}\left(f_{m},r,\theta ,t,x_{m}\right)\right)=\cos\left(2\pi f_{m}\left[t-\frac{r-x_{m}\sin\left(\theta \right)}{c}\right]\right), \end{array}$$

(21b) $$\begin{array}{cc} q_{m} & ≜\mathrm{imag}\left(h_{m}\left(f_{m},r,\theta ,t,x_{m}\right)\right)=\sin\left(2\pi f_{m}\left[t-\frac{r-x_{m}\sin\left(\theta \right)}{c}\right]\right), \end{array}$$

and weighting vector is defined as

(22a) $$\begin{array}{cc} \; & vs.≜\mathrm{real}(w(t)), \end{array}$$

(22b) $$\begin{array}{cc} \; & s≜\mathrm{imag}(w(t)). \end{array}$$

Thus, the signal $|h^{H}{\left(f,r,\theta ,t,x\right)w\left(t\right)|}^{2}$ can be derived as

(23) $$\begin{array}{cc} {\left|h^{H}\left(f,r,\theta ,t,x\right)w\left(t\right)\right|}^{2} & =k^{†}Ck+q^{†}Cq+2k^{†}Dq\\ \; & ≜y(k,q), \end{array}$$

where $C=vv^{†}+ss^{†}$, $D=vs^{†}-sv^{†}$, $k=[k_{1},k_{2},\ldots ,k_{M}]$, and $q=[q_{1},q_{2},\ldots ,q_{M}]$. To simplify the notations, we use $k_{u}$, $q_{u}$, $y_{u}\left(k_{u},q_{u}\right)$ to represent the corresponding variable of LU, and $k_{e}$, $q_{e}$, $y_{e}\left(k_{e},q_{e}\right)$ to represent the corresponding variable of Eve. Accordingly, the secrecy rate can be formulated as

(24) $$\begin{array}{cc} \Gamma (k_{u},q_{u},k_{e},q_{e})≜ & \log_{2}\left(1+\sigma_{u}^{-2}y_{u}\left(k_{u},q_{u}\right)\right)-\log_{2}\left(1+\sigma_{e}^{-2}y_{e}\left(k_{e},q_{e}\right)\right). \end{array}$$

As a result, for a given w and f, the {x}-subproblem can be expressed as

(25) $$\begin{array}{cc} \max_{x}\; & \Gamma (k_{u},q_{u},k_{e},q_{e})\\ s.t.\;\; & (6d)\;\mathrm{and}\;(6c)\;\mathrm{are}\;\mathrm{satisfied}. \end{array}$$

Notice that problem (25) remains non-convex because of the complexity of the objective function. In order to address the problem (25) and ensure that MA position solutions in each iteration are guaranteed to satisfy the constraint (6b) and (6c), we employ the PGA method to find a locally optimal solution [37]. Specifically, employing the PGA, the update rule for *x* is derived as

(26a) $$\begin{array}{cc} \; & x^{i+1}=x^{i}+\rho \nabla_{x^{i}}\Gamma \left(k_{u},q_{u},k_{e},q_{e}\right), \end{array}$$

(26b) $$\begin{array}{cc} \; & x^{i+1}=P\left\{x^{i+1},d,L\right\}, \end{array}$$

where the superscript denotes the iteration number, i.e., $x^{i}$ represents the antenna positions in *i*th iteration, and $x^{i+1}$ is in (i+1)th iteration. P represents the projection operation, which ensures that the constraints (6b) and (6c) are satisfied. $\nabla_{x^{i}}\Gamma \left(k_{u},q_{u},k_{e},q_{e}\right)$ denotes the gradient of $\Gamma (k_{u},q_{u},k_{e},q_{e})$ with respect to $x^{i}$. ρ is the step size for gradient ascent.

Then, we compute the gradient $\nabla_{x^{i}}\Gamma \left(k_{u},q_{u},k_{e},q_{e}\right)$, which is derived as

(27) $$\begin{array}{c} \nabla_{x^{i}}\Gamma \left(k_{u},q_{u},k_{e},q_{e}\right)=\frac{1}{\ln2}\times \left(\begin{array}{c} \frac{\sigma_{u}^{-2}\nabla_{x^{i}}y_{u}\left(k_{u},q_{u}\right)}{1+\sigma_{u}^{-2}y_{u}\left(k_{u},q_{u}\right)}-\frac{\sigma_{e}^{-2}\nabla_{x^{i}}y_{e}\left(k_{e},q_{e}\right)}{1+\sigma_{e}^{-2}y_{e}\left(k_{e},q_{e}\right)} \end{array}\right), \end{array}$$

where $\nabla_{x^{i}}y_{u}\left(k_{u},q_{u}\right)$ and $\nabla_{x^{i}}y_{e}\left(k_{e},q_{e}\right)$ are the gradient of $y_{u}\left(k_{u},q_{u}\right)$ and $y_{e}\left(k_{e},q_{e}\right)$ with respect to $x^{i}$, which can be derived as

(28a) $$\begin{array}{cc} \; & \nabla_{x_{m}^{i}}y_{u}\left(k_{u},q_{u}\right)=\frac{\partial y_{u}\left(k_{u},q_{u}\right)}{\partial k_{u,m}}\frac{\partial k_{u,m}}{\partial x_{m}}+\frac{\partial y_{u}\left(k_{u},q_{u}\right)}{\partial q_{u,m}}\frac{\partial q_{u,m}}{\partial x_{m}}\\ \; & =-2\pi f_{m}\frac{\sin(\theta_{u})}{c}\sin\left(2\pi f_{m}\left[t-\frac{r_{u}-x_{m}\sin\left(\theta_{u}\right)}{c}\right]\right)\frac{\partial y_{u}\left(k_{u},q_{u}\right)}{\partial k_{u,m}}\\ \; & \;\;\;+2\pi f_{m}\frac{\sin(\theta_{u})}{c}\cos\left(2\pi f_{m}\left[t-\frac{r_{u}-x_{m}\sin\left(\theta_{u}\right)}{c}\right]\right)\frac{\partial y_{u}\left(k_{u},q_{u}\right)}{\partial q_{u,m}}, \end{array}$$

(28b) $$\begin{array}{cc} \; & \nabla_{x_{m}^{i}}y_{e}\left(k_{e},q_{e}\right)=\frac{\partial y_{e}\left(k_{e},q_{e}\right)}{\partial k_{e,m}}\frac{\partial k_{e,m}}{\partial x_{m}}+\frac{\partial y_{e}\left(k_{e},q_{e}\right)}{\partial q_{e,m}}\frac{\partial q_{e,m}}{\partial x_{m}}\\ \; & =-2\pi f_{m}\frac{\sin(\theta_{e})}{c}\sin\left(2\pi f_{m}\left[t-\frac{r_{e}-x_{m}\sin\left(\theta_{e}\right)}{c}\right]\right)\frac{\partial y_{e}\left(k_{e},q_{e}\right)}{\partial k_{e,m}}\\ \; & \;\;\;+2\pi f_{m}\frac{\sin(\theta_{e})}{c}\cos\left(2\pi f_{m}\left[t-\frac{r_{e}-x_{m}\sin\left(\theta_{e}\right)}{c}\right]\right)\frac{\partial y_{e}\left(k_{e},q_{e}\right)}{\partial q_{e,m}}, \end{array}$$

based on which we denote

(29a) $$\begin{array}{cc} \; & Z_{u}=\mathrm{diag}\left({\left\{2\pi f_{m}\frac{\sin(\theta_{u})}{c}\sin\left(2\pi f_{m}\left[t-\frac{r_{u}-x_{m}\sin\left(\theta_{u}\right)}{c}\right]\right)\right\}}_{m=1}^{M}\right), \end{array}$$

(29b) $$\begin{array}{cc} \; & Z_{e}=\mathrm{diag}\left({\left\{2\pi f_{m}\frac{\sin(\theta_{e})}{c}\sin\left(2\pi f_{m}\left[t-\frac{r_{e}-x_{m}\sin\left(\theta_{e}\right)}{c}\right]\right)\right\}}_{m=1}^{M}\right), \end{array}$$

(29c) $$\begin{array}{cc} \; & G_{u}=\mathrm{diag}\left({\left\{\pi f_{m}\frac{\sin(\theta_{u})}{c}\cos\left(2\pi f_{m}\left[t-\frac{r_{u}-x_{m}\sin\left(\theta_{u}\right)}{c}\right]\right)\right\}}_{m=1}^{M}\right), \end{array}$$

(29d) $$\begin{array}{cc} \; & G_{e}=\mathrm{diag}\left({\left\{2\pi f_{m}\frac{\sin(\theta_{e})}{c}\cos\left(2\pi f_{m}\left[t-\frac{r_{e}-x_{m}\sin\left(\theta_{e}\right)}{c}\right]\right)\right\}}_{m=1}^{M}\right), \end{array}$$

where diag(·) denote the diagonal operation. And it is easy to verify that

(30a) $$\begin{array}{cc} \; & {\left[\frac{\partial y_{u}\left(k_{u},q_{u}\right)}{\partial k_{u,1}},\frac{\partial y_{u}\left(k_{u},q_{u}\right)}{\partial k_{u,2}},\ldots ,\frac{\partial y_{u}\left(k_{u},q_{u}\right)}{\partial k_{u,M}}\right]}^{†}=2Ck_{u}+2Dq_{u}, \end{array}$$

(30b) $$\begin{array}{cc} \; & {\left[\frac{\partial y_{u}\left(k_{u},q_{u}\right)}{\partial q_{u,1}},\frac{\partial y_{u}\left(k_{u},q_{u}\right)}{\partial q_{u,2}},\ldots ,\frac{\partial y_{u}\left(k_{u},q_{u}\right)}{\partial q_{u,M}}\right]}^{†}=2Cq_{u}-2Dk_{u}, \end{array}$$

(30c) $$\begin{array}{cc} \; & {\left[\frac{\partial y_{e}\left(k_{e},q_{e}\right)}{\partial k_{e,1}},\frac{\partial y_{e}\left(k_{e},q_{e}\right)}{\partial k_{e,2}},\ldots ,\frac{\partial y_{e}\left(k_{e},q_{e}\right)}{\partial k_{e,M}}\right]}^{†}=2Ck_{e}+2Dq_{e}, \end{array}$$

(30d) $$\begin{array}{cc} \; & {\left[\frac{\partial y_{e}\left(k_{e},q_{e}\right)}{\partial q_{e,1}},\frac{\partial y_{e}\left(k_{e},q_{e}\right)}{\partial q_{e,2}},\ldots ,\frac{\partial y_{e}\left(k_{e},q_{e}\right)}{\partial q_{e,M}}\right]}^{†}=2Cq_{e}-2Dk_{e}. \end{array}$$

Based on the above analysis, the following result is finally derived

(31a) $$\begin{array}{cc} \nabla_{x^{i}}y_{u}\left(k_{u},q_{u}\right) & =-Z_{u}\left(2Ck_{u}+2Dq_{u}\right)+G_{u}\left(2Cq_{u}-2Dk_{u}\right), \end{array}$$

(31b) $$\begin{array}{cc} \nabla_{x^{i}}y_{e}\left(k_{e},q_{e}\right) & =-Z_{e}\left(2Ck_{e}+2Dq_{e}\right)+G_{e}\left(2Cq_{e}-2Dk_{e}\right). \end{array}$$

Next, $\nabla_{x^{i}}\Gamma \left(k_{u},q_{u},k_{e},q_{e}\right)$ can be computed as

(32) $$\begin{array}{c} \nabla_{x^{i}}\Gamma \left(k_{u},q_{u},k_{e},q_{e}\right)=\frac{1}{\ln2}\times \left(\begin{array}{c} \frac{\sigma_{u}^{-2}\left(-Z_{u}\left(2Ck_{u}+2Dq_{u}\right)+G_{u}\left(2Cq_{u}-2Dk_{u}\right)\right)}{1+\sigma_{u}^{-2}y_{u}\left(k_{u},q_{u}\right)}\\ -\frac{\sigma_{e}^{-2}\left(-Z_{e}\left(2Ck_{e}+2Dq_{e}\right)+G_{e}\left(2Cq_{e}-2Dk_{e}\right)\right)}{1+\sigma_{e}^{-2}y_{e}\left(k_{e},q_{e}\right)} \end{array}\right). \end{array}$$

To guarantee that the MA positions obtained in each iteration consistently satisfy the constraints (6b) and (6c), we employ the projection operation after performing gradient ascent. Without loss of generality, we assume that $0\leq x_{1}\leq x_{2}\leq \ldots \leq x_{M}\leq L$. Then, according to constraint (6b), we have $x_{2}-x_{1}\geq d_{min},\ldots ,x_{M-1}-x_{M-2}\geq d_{min},x_{M}-x_{M-1}\geq d_{min}$. It is easy to verify that

(33) $$\begin{array}{c} x_{N}\geq x_{N-1}+d_{min}\geq \ldots \geq x_{1}+\left(M-1\right)d_{min}. \end{array}$$

Furthermore, considering the constraint (6c), we have $x_{m}+\left(M-m\right)d_{min}\leq L,m=1,2,\ldots ,M$. As a result, the feasible range for each $x_{m}$ can be expressed as

(34a) $$\begin{array}{cc} \; & x_{1}\in \left[0,L-\left(M-1\right)d_{min}\right], \end{array}$$

(34b) $$\begin{array}{cc} \; & x_{m}\in \left[x_{m-1}+d_{min},L-\left(M-m\right)d_{min}\right],m=2,\ldots ,M. \end{array}$$

In summary, the projection function is

(35) $$\begin{array}{c} \begin{array}{c} P\left\{x^{i+1},d_{min},L\right\}:\left\{\begin{array}{c} x_{1}^{i+1}=\max\left(0,\min\left(L-\left(M-1\right)d_{min},x_{1}^{i+1}\right)\right),\\ x_{m}^{i+1}=\max\left(x_{m-1}^{i+1}+d_{min},\min\left(L-\left(M-m\right)d_{min},x_{m}^{i+1}\right)\right),\\ \;\;\;\;\;\;\;\;\;\;\;\;\;\;\;\;\;\;\;\;\;\;\;\;\;\;\;\;\;\;\;\;\;\;\;\;\;\;\;\;\;\;\;m=2,3,\ldots ,M. \end{array}\right. \end{array} \end{array}$$

Utilizing $\nabla_{x^{i}}\Gamma \left(k_{u},q_{u},k_{e},q_{e}\right)$ and $P\{x^{i+1},d_{min},L\}$, the PGA method iteratively updates $x^{i}$ according to Equations (26a) and (26b) until problem (25) converges to a constant value.

### 3.3. {w}-Subproblem

We update the beamformer w with given x and f

(36a) $$\begin{array}{cc} \max_{w}\; & R_{\sec}\left(w\right)\\ s.t.\;\; & (6e)\;\mathrm{is}\;\mathrm{satisfied}. \end{array}$$

The {w}-subproblem can be rewritten as

(37a) $$\begin{array}{cc} \max_{w}\; & \frac{1+w^{H}Aw}{1+w^{H}Ow} \end{array}$$

(37b) $$\begin{array}{cc} s.t.\;\; & {∥w∥}_{2}^{2}=P, \end{array}$$

where $A=\sigma_{u}^{-2}h_{u}\left(f,t,x\right)h_{u}^{H}\left(f,t,x\right)$ and $O=\sigma_{e}^{-2}h_{e}\left(f,t,x\right)h_{e}^{H}\left(f,t,x\right)$.

**Proposition** **1.** *We propose that* (37a)*,* (37b) *and* (36a) *are equivalent.*

**Proof.**  The proof is relegated to Appendix A.    □

The optimal solution can be calculated as [36]

(38) $$w^{*}=\sqrt{P}E_{max},$$

where $E_{max}$ is the normalized eigenvector associated with the largest eigenvalue of the matrix ${\left(O+\frac{1}{P}I_{N}\right)}^{-1}\left(A+\frac{1}{P}I_{N}\right)$, $I_{N}$ represents the N×N identity matrix, and ${\left(\cdot \right)}^{-1}$ denotes the inverse operation.

### 3.4. Algorithm Summary

The AO algorithm to problem (6a)–(6e) is summarized as Algorithm 1. For the *i*th iteration, the complexity is detailed as follows:

- $\left\{f_{m}\right\}$-subproblem: O(2M).
- {x}-subproblem: the complexity of gradient computation and projection are $O(M^{2})$ and O(M), respectively.
- {w}-subproblem: $O(M^{3})$.

As a result, the per-iteration computational complexity of Algorithm 1 is $O(2\left(s_{1}+1\right)M+\left(s_{2}+1\right)\left(M^{2}+M\right)+M^{3})$.

**Proposition** **2.** *Each step of Algorithm 1 generates a stationary point.*

**Proof.**  The proof is relegated to Appendix B.    □

**Algorithm 1** AO approach for solving problem (6a)–(6e)
**Initialize:** $f^{0}$, $x^{0}$, $w^{0}$, i=0.  1:**Repeat**:  2:i=i+1, $s_{1}=0$, $s_{2}=0$.
**Updating {f}**  3:      **Repeat**:  4:      $s_{1}=s_{1}+1$, $m=(s_{1}\;\mathrm{mod}\;M)+1$.  5:      Compute $\left\{\kappa_{m,n,k},\zeta_{m,n,k},\delta_{m,n,k}\right\}$ according to (18a) and (18b).  6:      $f_{m}^{s_{1}}\leftarrow {\left[\frac{\sum_{\begin{array}{c} n=1\\ n\neq m \end{array}}^{M}\kappa_{m,n}\zeta_{m,n}}{\sum_{\begin{array}{c} n=1\\ n\neq m \end{array}}^{M}\kappa_{m,n}}\right]}_{f_{c}-\Delta F}^{f_{c}+\Delta F}$.  7:      $f_{n}^{s_{1}}\leftarrow f_{n}^{s_{1}-1},\;\;\forall \;n\neq m$.  8:      **Until converge**  9:      $\left\{f^{i}\right\}=\left\{f^{s_{1}}\right\}$.
**Updating {x}**10:      **Repeat**:11:      $s_{2}=s_{2}+1$.12:      Compute $\nabla_{x^{s_{2}}}\Gamma \left(k_{u},q_{u},k_{e},q_{e}\right)$ according to (32).13:      Update $\left\{x^{s_{2}}\right\}$ according to (26a) and (26b)14:      **Until converge**15:      $\left\{x^{i}\right\}=\left\{x^{s_{2}}\right\}$
**Updating {w}**16:      Optimize the beamformer $w^{i}$ by (38).17:
**Until converge**
**Output:** 

$R^{★}$




## 4. Numerical Simulations

In this section, we present numerical simulations to validate the effectiveness of our proposed MA-FDA approach in enhancing the secrecy performance. We validate the superiority of the proposed FDA approach over the PA approach and FPA schemes that lack optimized frequency offsets or antenna positions. These benchmark schemes include the following: (1) FPA with frequency offsets optimization (op-FDA), where the antenna positions is uniform linear distribution, i.e., $x_{m}=\left(m-1\right)d,\forall m$; (2) MA with random frequency offsets within the range of [−ΔF,ΔF]; (3) MA-PA approach; and (4) traditional PA approach, i.e., FPA-PA approach.

Unless stated otherwise, the following simulations assume that the carrier frequency is $f_{c}=1$ GHz, the maximum frequency offsets is $\Delta F=10^{-5}f_{c}=10$ KHz, and the coverage area of the MA array is [0,L]=[0,20d]. The noise power is set as $\sigma_{u}^{2}=\sigma_{e}^{2}=1$ to normalize the power of large-scale channel fading. The positions of LU and Eve are given by $\left(r_{u},\theta_{u}\right)=(1000$ m, $30^{\circ })$ and $\left(r_{e},\theta_{e}\right)=(1000$ m, $32^{\circ })$, respectively. Additionally, we set the step size for the PGA method as ρ=0.01, and the PGA stopping criterion as $\Gamma \left(k_{u}^{i},q_{u}^{i},k_{e}^{i},q_{e}^{i}\right)-\Gamma \left(k_{u}^{i-1},q_{u}^{i-1},k_{e}^{i-1},q_{e}^{i-1}\right)\leq \mu$, and set $\mu =10^{-3}$. The BSUM stopping criterion is set as $|h_{e}^{†}\left(f^{i},t\right)h_{u}\left(f^{i},t\right){|}^{2}-{\left|h_{e}^{†}\left(f^{i-1},t\right)h_{u}\left(f^{i-1},t\right)\right|}^{2}\leq \mu$. The termination condition of Algorithm 1 is $R_{sec}^{i}-R_{sec}^{i-1}\leq \varepsilon$, and $\varepsilon =10^{-3}$.

**Figure 3:**
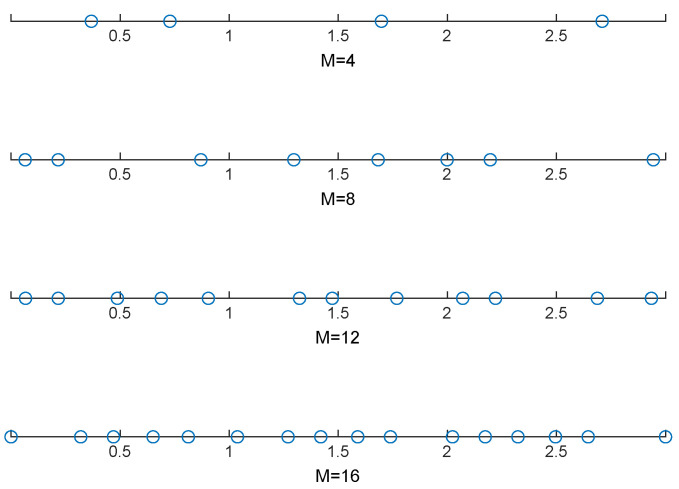
Optimized positions of the MA array.

Figure 3 illustrates the optimized positions of the MA array. It can be observed that, as opposed to the FPA array with immovable positions given by $x_{\mathrm{FPA}}={\left[0,d,\ldots ,\left(M-1\right)d\right]}^{†}$, where $d=c/(2f_{c})=0.15$ m, the spatial distribution of the MA array deviates from a simple uniform pattern. Although the movement region is larger for the MA array, it is crucial to recognize that the signal wavelength λ is quite small, particularly in mmWave frequency bands. As a result, the increase in distance is small, suggesting that the additional manufacturing costs are insignificant or even negligible.

**Figure 4:**
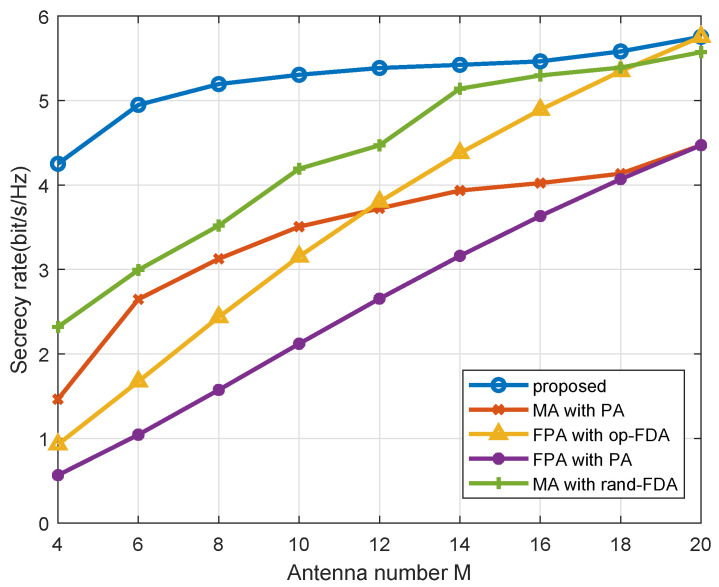
Secrecy rate performance across different antenna number *M*.

Figure 4 shows the relationship between the secrecy rate and the antenna number *M*. It can be observed that a larger *M* results in higher secrecy rate, due to the improved angle discrimination capability of the array. Furthermore, the proposed MA-FDA approach outperforms other methods. In addition, when *M* is small, i.e., the array minimum range requirement (M−1)d is smaller than *L*, and the mobility of the antenna is fully utilized. Consequently, the MA approaches outperform the FPA approaches. As the number of antennas *M* increase, the secrecy rates of FPA and MA approaches are becoming increasingly similar. And when *M* increases so that *L* is equal to the minimum required range for the antennas, the secrecy rates of FPA and MA are the same, since the mobility of the antenna can not be fully utilized.

**Figure 5:**
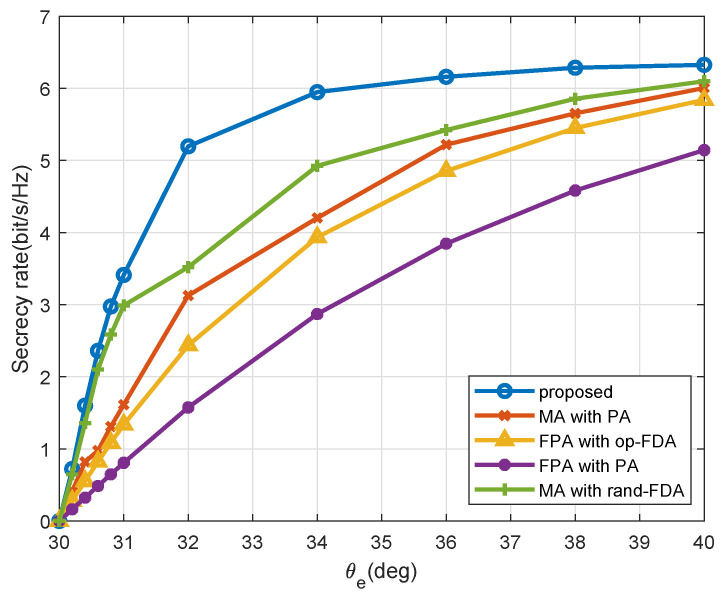
Secrecy rate performance across different Eve angle $\theta_{e}$.

Figure 5 shows a comparison of the secrecy rates for various Eve angles $\theta_{e}$. We fix $r_{u}=r_{e}=1000m$, $\theta_{u}=30^{\circ }$. When $\theta_{e}=\theta_{u}=30^{\circ }$, $h_{u}\left(f,t,x\right)$ and $h_{e}\left(f,t,x\right)$ exhibit linear correlation regardless of the value of f. Consequently, neither the FDA nor the conventional PA approach ensures PHY security. As $\theta_{e}$ moves away from $\theta_{u}$, the effectiveness of both approaches begins to improve. Notably, the MA-FDA beamforming method surpasses the other approaches when $\theta_{e}\neq \theta_{u}$. This enhanced performance is due to the optimization of frequency offsets and antenna positions, which effectively decouples the channels of the LU and Eve, regardless of their close positioning, thereby enhancing the secrecy rate.

**Figure 6:**
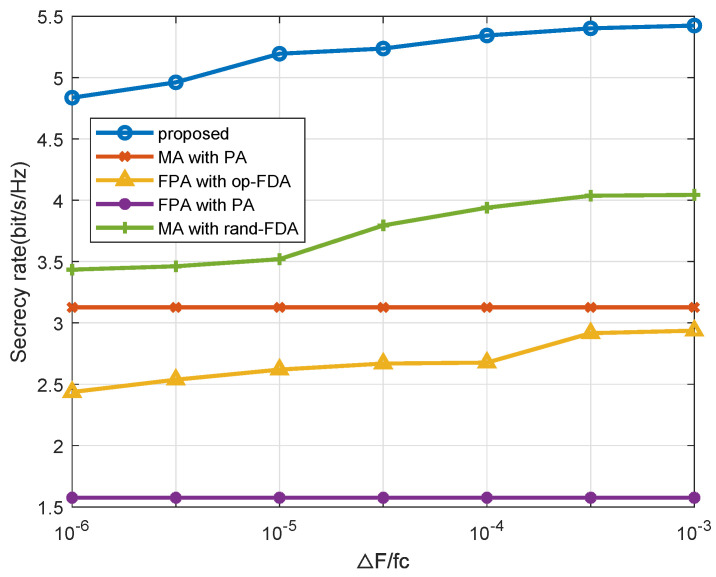
Secrecy rate performance across different frequency offset upper bounds ΔF.

Figure 6 provides a comparison of secrecy rates for the different upper bounds of frequency offset ΔF. The results indicate that the secrecy rate of the PA approach remains unchanged with different ΔF. In contrast, the secrecy rates of all FDA approaches increase as the frequency offset upper bound grows. However, achieving a large ΔF in practical applications is challenging owing to the necessity of phase coherence, gain anti-degradation, and signal decorrelation. Fortunately, the proposed MA-FDA approach still achieves strong performance even with a relatively small ΔF, such as $\Delta F=10^{-5}f_{c}$.

**Figure 7:**
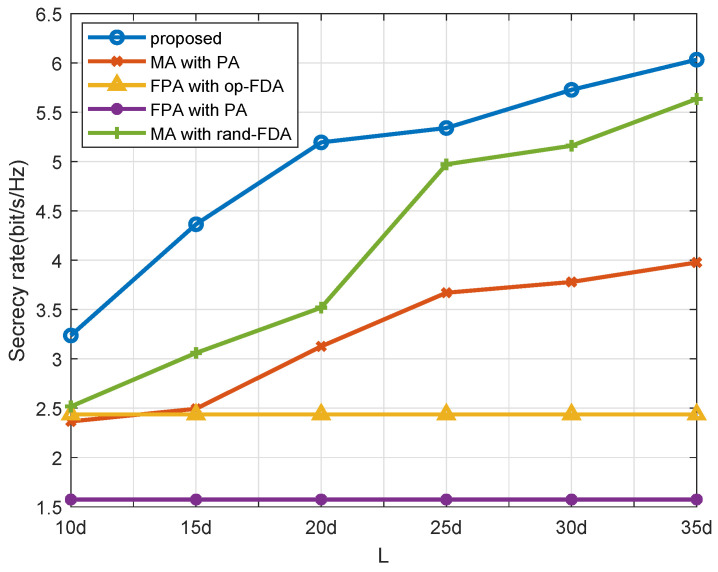
Secrecy rate performance across different antenna range *L*.

Figure 7 depicts a comparison of secrecy rates for various antenna movement upper bound *L*. Notably, the FPA method is independent of *L*. In addition, the movement upper bound *L* constrains the antenna mobility in the MA approach. As *L* increases, the mobility of MA is improved. However, a larger *L* incurs additional costs. Therefore, *L* cannot be increased indefinitely, and it is necessary to select *L* reasonably to balance the cost and effectiveness.

**Figure 8:**
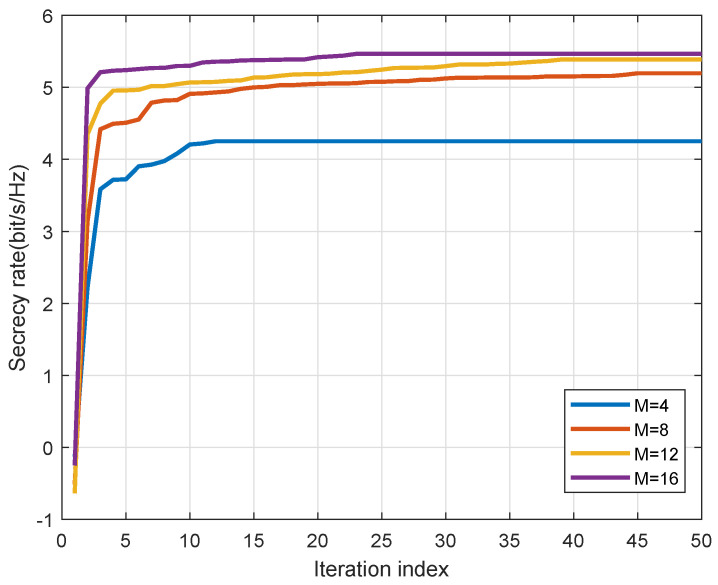
Typical converging traces of AO algorithm.

The convergence performance of the AO algorithm is illustrated in Figure 8. Notably, the objective in the outer layer exhibits rapid increases in the first few iterations, converging to a constant without exceeding 25 iterations in all cases, demonstrating the computational efficiency of the proposed algorithms.

## 5. Conclusions

In this paper, we propose a novel approach to improve PHY security through multidimensional channel reconstruction. The main idea is to employ MA and FDA technologies within the system and jointly optimize the antenna positions of the MA, FDA frequency offsets and beamformer to decouple the channels of LU and Eve. We first formulate a secrecy rate maximization problem subject to physical and energy constraints. Then, we propose an AO algorithm that employs PGA and BSUM method to iteratively update parameters, and leads to a stationary solution as the final outcome. Simulation results demonstrate that the MA-FDA approach outperforms other related schemes that use only FDA or MA technology.

## Data Availability

Data are contained within the article.

## Abbreviations

The following abbreviations are used in this manuscript:

PHY	Physical-Layer
LU	Legitimate User
Eve	Eavesdropper
BS	Base Station
AO	Alternating Optimization
PGA	Projected Gradient Ascent
BSUM	Block Successive Upper-Bound Minimization
FDA	Frequency Diverse Arrays
MA	Movable Antenna
PA	Phased Array
SINR	Signal-to-Interference-Plus-Noise Ratio
FPA	Fixed Position Antenna

## Appendix A. Proof of Proposition 1

Since the logarithmic function is monotonic, it is straightforward to demonstrate that maximizing (36a) is equivalent to maximizing (37a). Next, we prove that the optimal solution is attained when constraint (6e) holds with equality, i.e., ${∥w∥}_{2}^{2}=P$. We prove it by contradiction. Define $a\left(w\right)\overset{\Delta }{\;=}w^{H}Aw$ and $o\left(w\right)\overset{\Delta }{\;=}w^{H}Ow$. It is easy to verify that

(A1) $$\begin{array}{cc} \frac{1+a(w)}{1+o(w)}\; & =\frac{1+o(w)+(a(w)-o(w))}{1+o(w)}\\ \; & =1+\frac{a(w)-o(w)}{1+o(w)}. \end{array}$$

Suppose the optimal solution is $∥w^{*}{∥}_{2}^{2}<P$, let $w=\ell w^{*},\ell >1$, and ${∥w∥}_{2}^{2}\leq P$, the objective value is

(A2) $$\begin{array}{cc} 1+\frac{a(\ell w)-o(\ell w)}{1+o(\ell w)} & =1+\frac{\ell^{2}a\left(w\right)-\ell^{2}o\left(w\right)}{1+\ell^{2}o\left(w\right)}\\ \; & >1+\frac{\ell^{2}a\left(w\right)-\ell^{2}o\left(w\right)}{\ell^{2}+\ell^{2}o\left(w\right)}\\ \; & =1+\frac{a(w)-o(w)}{1+o(w)}. \end{array}$$

As a result, if $∥w^{*}{∥}_{2}^{2}<P$, we can increase w to improve the objective value. Therefore, the equalities in constraints (6e) must hold at the optimal solution of problem (36a), otherwise we can increase w to improve the objective value. Hence, (37a), (37b) and (36a) are equivalent.

## Appendix B. Proof of Proposition 2

$w^{★}$ is the optimal solution of the w-subproblem. In the following, we primarily analyze the convergence of the updates for the f-subproblem and x-subproblem.

According to (17a)–(17e), the convex quadratic function is designed to satisfy three key conditions: (i) concavity, (ii) adherence to the BSUM assumption, including tightness, identical-derivative, and continuity; and (iii) uniqueness of the solution. Additionally, the regularity condition holds naturally due to the smoothness of $g_{m}\left(f_{m};f_{-m}^{i-1}\right)$, while the upper bound condition can be verified by comparing derivatives. As a result, the requirements of [[38], Theorem 2] are all satisfied, ensuring that every limit point of the iterates produced by the BSUM algorithm is a stationary point.

Since the x-subproblem (25) is smooth and the gradient of $\Gamma (k_{u},q_{u},k_{e},q_{e})$ is Lipschitz-continuous, applying the optimality condition of the projection yields:

(A3) $$\Gamma^{s_{2}+1}\leq \Gamma^{s_{2}}-\beta {∥\nabla \Gamma^{s_{2}}∥}^{2},$$

where β>0. This demonstrates that the objective function value decreases monotonically, i.e.,

(A4) $$\Gamma^{1}>\Gamma^{2}>\ldots >\Gamma^{★},$$

where $\Gamma^{★}$ is a lower bound of Γ. Since Γ decreases monotonically and has a lower bound, it follows that

(A5) $$\lim_{s_{2}\to \infty }\left(\Gamma^{s_{2}+1}-\Gamma^{s_{2}}=0\right),$$

combined with (A3), we have

(A6) $$\lim_{s_{2}\to \infty }\left(\nabla \Gamma^{s_{2}+1}=0\right),$$

This implies that the algorithm eventually converges to a stationary point.
